# Anti-programmed cell death protein 1 inhibitor induced squamoproliferative eruption: A rare side effect

**DOI:** 10.1016/j.jdcr.2026.07.015

**Published:** 2026-07-20

**Authors:** Melissa Crous, Li Jie Helena Yoo, Marion Leahy, Bianca Mostert, Kashif Ahmad

**Affiliations:** Department of Dermatology, University Hospital Limerick, Co. Limerick, Ireland

**Keywords:** acitretin, anti-PD 1 inhibitor, melanoma, side effects, squamoproliferative eruption

## Case description

An 82-year-old male, retired engineer, with stage 3C BRAF wild type melanoma presented with multiple hyperkeratotic nodules and plaques on an erythematous base affecting his extremities (Fig 1, *A* and *B*) as well as linear keratotic papules noted on the palms (Fig 1, *C*). Preceding the rash, the patient completed 5 cycles of 200 mg pembrolizumab every 3 weeks, an anti-programmed death 1 inhibitor. He was not on a concomitant MEK/BRAF inhibitor, and the patient had no significant history of sun exposure. He developed significant generalized pruritus after cycle 2 which was managed with antihistamines and topical hydrocortisone. The rash appeared after cycle 5. His oncology history includes a nodular melanoma, Breslow thickness 6 mm without ulceration excised from the left pretibial area. Wide local excision and sentinel node biopsy were clear. He subsequently developed pigmented macules around the primary surgical site (Fig 2), which were confirmed as melanoma in-transit microsatellite lesions. Staging PET computed tomography and magnetic resonance imaging brain showed no metastasis. His medical history includes stable chronic oral lichen planus, hypertension, type 2 diabetes mellitus and benign prostatic hyperplasia. His regular medications include dapagliflozin, dutasteride, metformin, bisoprolol, ramipril, and doxazosin. Skin biopsies for rash evaluation were taken from his scalp, left wrist, and forearm nodules. These demonstrated moderately differentiated squamous cell carcinomas. A diagnosis of anti-programmed death 1 inhibitor-induced squamoproliferative eruption was made, and pembrolizumab was stopped. Different treatment modalities were utilized, including excision, cryotherapy, and acitretin. He completed 1 year of follow up with oncology with all surveillance imaging being clear to date. He is now discharged from the oncology service and under regular follow up with dermatology.

**Fig 1:**
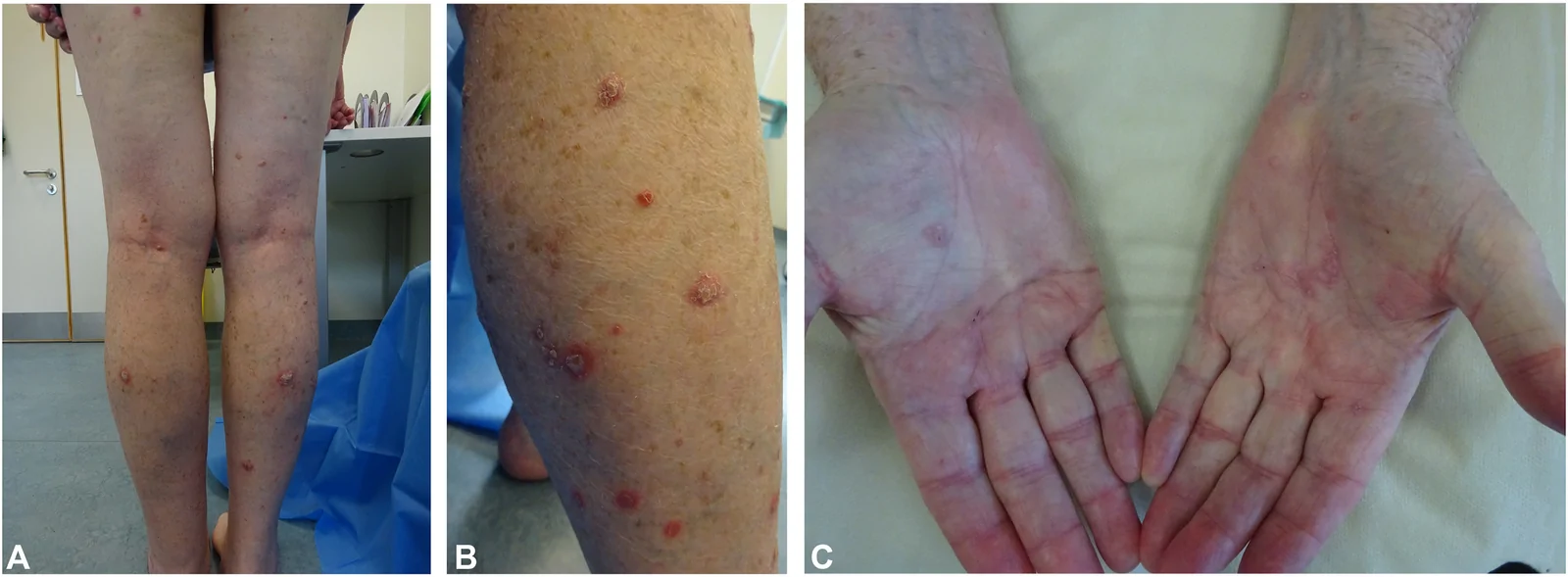
Generalised papulosquamous eruption on seen on the limbs (**A, B**) and palms (**C**).

**Fig 2:**
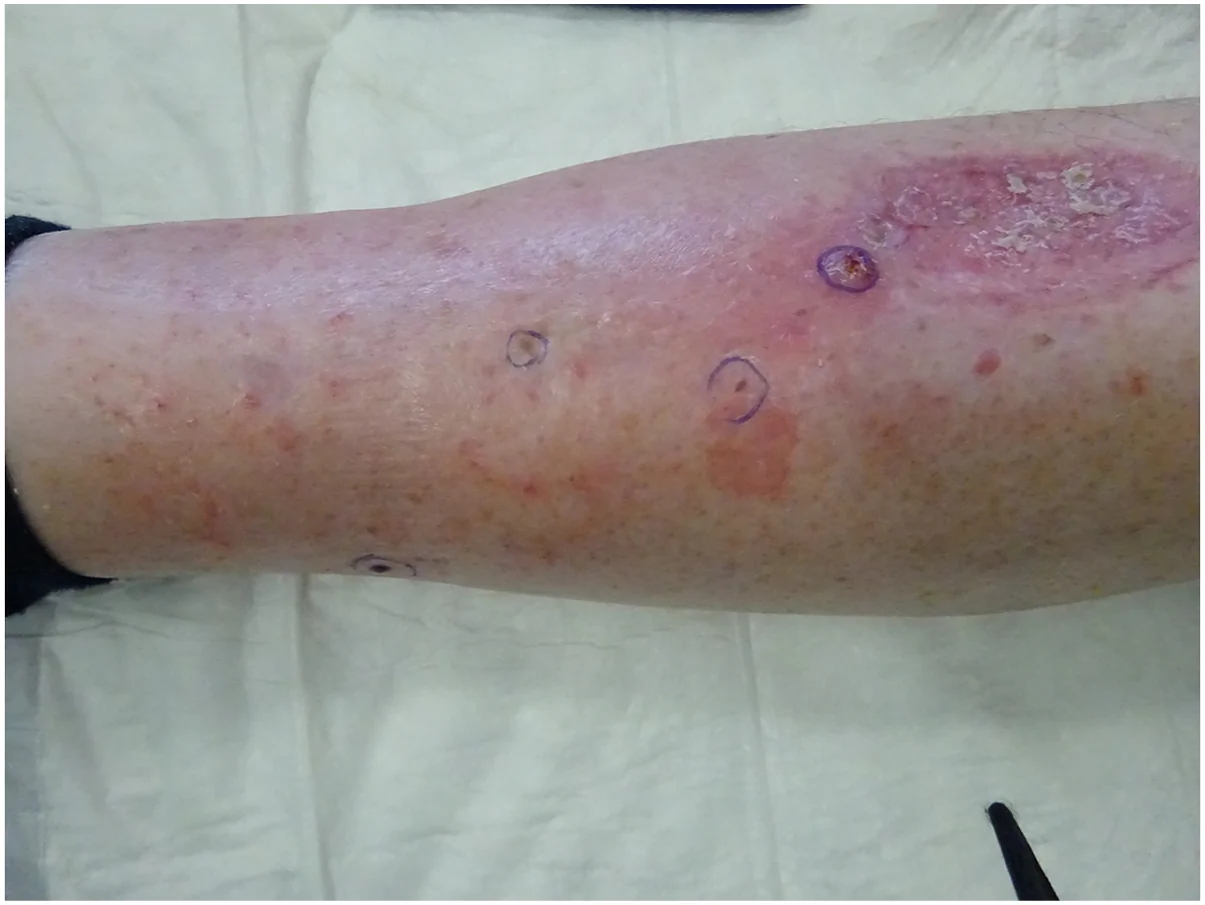
In-transit microsatelite lesions seen around the primary surgical site of the excised nodular melanoma.

**Question: Which of the following actions is recommended in a squamoproliferative eruption secondary to pembrolizumab in a patient with asymptomatic inflammatory plaques?**

**A.** Stop the drug; wait for the rash to resolve before recommencement.
**B.** Continue the drug; advice patient that the lesions might progress.
**C.** Continue the drug; commence localized skin treatment.
**D.** Stop the drug; commence topical treatment and acitretin.
**E.** Add cemiplimab.

Answer: C.

## Discussion

Cutaneous adverse events from pembrolizumab are well-described. Although the incidence of a cutaneous adverse reaction has been reported in 44%,^1^ the development of multiple keratoacanthoma and squamous cell carcinoma is rare. This is counterintuitive especially when it is used to treat metastatic cutaneous squamous cell carcinoma. It is hypothesized that this phenomenon is driven by a local cutaneous immunosuppressed tumor microenvironment unmasking previously actinically damaged keratinocytes.^2^ This case highlights pembrolizumab-induced eruptive squamoproliferative lesions in photo-exposed sites of actinically damaged skin in a patient with in-transit melanoma.

The onset of the eruptive keratoacanthoma are typically 19.8 weeks after initiation of treatment, affecting older patients (median age of 76.4) with rapidly growing karatoacanthomas.^1^ These clinical features are like our patient.

To date, treatment options are limited to case reports and include potent topical steroids, nicotinamide, surgical excision, and acitretin.^2^ A case series (*n* = 7) in 2022 suggested that acitretin, ranging from a dose of 10 mg to 50 mg depending on disease severity, achieved skin clearance in the majority of patients (85%, *n* = 6) by 21 weeks.^1^ After 8 weeks of 20 mg acitretin, our patient experienced skin clearance. The American Society of Clinical Oncology, suggest categorizing patients into mild, moderate or severe disease based on the body surface area (BSA) involved. In the case of mild disease (BSA <10%), which is predominantly inflammatory and asymptomatic, immunotherapy can be continued alongside topical steroids or curettage. In the case of moderate disease (BSA 10% to 30%), immunotherapy should be held temporarily and acitretin commenced if no contraindication. Severe disease (BSA >30%) with proven cutaneous malignancy requires excision and discontinuation of immunotherapy.^2^^,^^3^

The incidence of melanoma is increasing, and the use of anti-programmed death 1 inhibitors has become more common. This case highlights the clinical presentation, histology and response to acitretin of this rare but serious adverse event.

## Conflicts of interest

None disclosed.
